# Anti-*Toxocara canis* seroreactivity across autoimmune rheumatic diseases with exploratory IgG4 and eosinophil-associated signals in systemic lupus erythematosus: a cross-sectional case-control study

**DOI:** 10.1007/s00296-026-06249-3

**Published:** 2026-07-16

**Authors:** Lara Cardoso Rabello, Vinícius Campos Miranda, Thayná Souza de Paula, Isadora Aparecida Ferreira Silva, Laura Vitória Oliveira Lima, Bruna Matias Scucuglia, Mariângela de Lima Alves, Humberto Machado Resende, Roberto Ranza, Natália Berne Pinheiro, Luciana Farias Costa de Ávila, Raphael Chagas Silva, Cristiane Guimarães Morais, Carina da Silva Pinheiro, Rodrigo Rodrigues Cambraia-Miranda

**Affiliations:** 1 Laboratory of Diagnosis, Epidemiology and Control of Helminths (LADECH), Department of Parasitology, Institute of Biomedical Sciences (ICBIM), Federal University of Uberlândia (UFU), Uberlândia, MG Brazil; 2 Graduate Program in Applied Immunology and Parasitology (PPGIPA), Institute of Biomedical Sciences (ICBIM), Federal University of Uberlândia (UFU), Uberlândia, MG Brazil; 3 Rheumatology Sector, Hospital de Clínicas, Federal University of Uberlândia (HC- UFU), Uberlândia, MG Brazil; 4 Department of Microbiology and Parasitology, Institute of Biology, Federal University of Pelotas (UFPel), Pelotas, RS Brazil; 5 Laboratory of Allergy and Acarology, Institute of Health Sciences, Federal University of Bahia (UFBA), Salvador, BA Brazil; 6 Federal Institute of Education, Science and Technology of Triângulo Mineiro (IFTM), Uberaba, MG Brazil; 7 Faculty of Medicine, Laboratory of Parasitology, Federal University of Rio Grande (FURG), Rio Grande, RS Brazil

**Keywords:** Toxocariasis, Autoimmune diseases, Rheumatic diseases, Systemic lupus erythematosus, Immunoglobulin G, Eosinophils

## Abstract

Helminth-related immune responses may intersect with autoimmune rheumatic diseases, but disease-specific patterns remain incompletely understood. This study aimed to investigate anti-*Toxocara canis* seroreactivity and its immunological and clinical correlates in rheumatoid arthritis, systemic lupus erythematosus, and spondyloarthritis. In this cross-sectional case-control study, 442 individuals were evaluated across disease-specific comparisons. Serology followed a sequential strategy using native *T. canis* excretory-secretory antigen for initial IgG screening and a recombinant chimeric antigen for confirmatory IgG, IgG1, and IgG4 assessment. Cytokines, eosinophil counts, socioeconomic variables, disease activity, and functional disability were analyzed using adjusted regression models. Total anti-*T. canis* IgG seroreactivity was frequent across groups. Higher adjusted point estimates were observed in rheumatoid arthritis, systemic lupus erythematosus, and spondyloarthritis. SLE was the only disease group showing a nominally significant association with IgG seroreactivity compared with its respective control group (OR 2.44, 95% CI 1.16–5.14), although no serological association remained significant after FDR correction. SLE also showed a nominal signal of lower IgG4 seroreactivity than controls in the standard adjusted model (OR 0.20, 95% CI 0.04–0.99), but this finding did not remain significant after FDR correction or in small-cell sensitivity analyses. Higher eosinophil counts were associated with lower SLEDAI scores in negative binomial models (IRR per 100 eosinophils/mm³: 0.56, 95% CI 0.36–0.86), and this association persisted after adjustment for medication use, although it should be interpreted as exploratory given the low SLEDAI distribution and limited number of active disease events. Anti-*T. canis* seroreactivity was frequent across autoimmune rheumatic diseases and showed disease-specific exploratory patterns. In SLE, higher total IgG seroreactivity coexisted with a cautious, hypothesis-generating IgG4 signal, while eosinophil counts showed an exploratory independent association with lower SLEDAI scores. These findings suggest that helminth-related immune signatures may vary across autoimmune disease contexts and should be interpreted as exploratory associations rather than evidence of active infection or causal immunomodulation.

**Supplementary Information:**

The online version contains supplementary material available at 10.1007/s00296-026-06249-3.

## Introduction

Helminth infections and autoimmune diseases have often been conceptualized as immunologically contrasting conditions. Through co-evolution with humans, helminths tend to promote a regulatory immune environment. Early anti-helminth responses are typically dominated by type 2 immunity, with eosinophilia and increased IgE and IgG1 production, whereas persistent exposure tends to favor a modified type 2/regulatory milieu characterized by alternatively activated macrophages (M2), regulatory T- and B-cell responses, and increased interleukin-10 (IL-10), transforming growth factor-β (TGF-β), and IgG4 production [1–3]. By contrast, autoimmune diseases arise from the breakdown of immune tolerance and persistent inflammation. In this context, the hygiene hypothesis and its contemporary extensions, including the “old friends” hypothesis and microbiome-related concepts, propose that the loss of ancestral exposure to immunoregulatory organisms and disruption of host–microbiome interactions may contribute to the rising incidence of autoimmune disorders in industrialized settings [4, 5].

*Toxocara* spp. infection is a neglected zoonotic helminthiasis of global importance [6–8]. Human infection occurs through ingestion of embryonated eggs from contaminated environments or, less commonly, through consumption of raw or undercooked tissues from paratenic hosts or contaminated vegetables [9]. Humans are accidental hosts in whom larvae hatch in the intestine, penetrate the mucosa, enter the circulation, and migrate through somatic tissues without developing into adult worms. Although many infections remain asymptomatic or present with non-specific manifestations, this larval migration may result in clinically recognized syndromes such as visceral larva migrans, ocular larva migrans, covert toxocariasis, and neurotoxocariasis [10]. Because the parasite does not complete its life cycle in humans, eggs are not shed in stool, making coprological diagnosis uninformative. As a result, diagnosis relies largely on serology, although assays based on native antigens may show cross-reactivity with other helminth infections [11].

Meta-analyses estimate a global seroprevalence of approximately 19%, suggesting that nearly 1.4 billion individuals have been exposed worldwide [12]. In Brazil, reported seroprevalence rates vary widely, ranging from 4.2% to 65% depending on the population studied [13], and may be even higher in socially vulnerable settings, including indigenous communities [14]. Following infection, migrating larvae and their excretory-secretory antigens (TES) induce a predominantly type 2 immune response characterized by eosinophilia, Th2-associated cytokines, and parasite-specific antibody production. In human toxocariasis, this humoral response is usually dominated by IgG1, with significant IgM and IgE reactivity, whereas IgG4 appears less prominent overall, although it has been reported in some active cases [15]. In experimental models, this response can be granulomagenic and pathogenic [16, 17]. More broadly, chronic helminth infections have been associated with a more regulated immune profile [18], including immunoregulatory pathways linked to IgG4 production [19–21], supporting the biological plausibility of evaluating IgG4 as a qualitative, although indirect, marker of regulatory-associated features within anti-helminth immunity [22].

The relationship between helminth infections and systemic lupus erythematosus (SLE) is particularly complex. Experimental studies and review data suggest that helminth-derived immunomodulation may delay lupus-like disease onset and attenuate nephritis in murine models, at least partly through regulatory pathways [23]. In humans, however, the available evidence remains limited and sometimes conflicting. Although some observations are compatible with disease-attenuating effects in specific settings, an isolated case report has suggested that toxocariasis may clinically overlap with, or possibly contribute to the emergence of, lupus manifestations in susceptible individuals [24]. Recent review data further emphasize that *T. canis* may exert context-dependent effects in autoimmunity, with live infection potentially amplifying autoreactive pathways while defined parasite-derived molecules may display selective immunomodulatory properties [25]. Together, these observations highlight the need to investigate not only helminth exposure itself, but also the qualitative profile of the host immune response.

The interplay between helminth-driven immunoregulation and autoimmune rheumatic diseases (ARDs) remains unresolved. Although helminth-related interventions can ameliorate arthritis in experimental models [26], the qualitative profile of anti-helminth immunity in human autoimmunity remains poorly understood. SLE is characterized by B-cell hyperactivity and polyclonal antibody production [27], yet it is unclear how regulatory-associated components of the anti-helminth response, particularly IgG4, are expressed in this pro-inflammatory milieu. Whether similar alterations occur in rheumatoid arthritis (RA) and spondyloarthritis (SpA) also remains unknown. Addressing this question requires serological strategies that combine broad screening with more specific downstream characterization, since native antigens may generate cross-reactivity and obscure true response patterns [11].

In this study, we investigated the qualitative profile of anti-*T. canis* humoral responses in patients with ARDs and examined their exploratory associations with serum cytokine measurements, eosinophil counts, and clinical outcomes. Serological assessment followed a sequential strategy, with initial screening using native TES antigen and subsequent evaluation of TES-reactive samples using a recombinant chimeric antigen selected to improve specificity and reduce cross-reactivity [28].

## Materials and methods

### Study design, population, and ethics

We conducted a cross-sectional case-control study including 442 individuals. Participants were recruited and biological samples were collected between February 2023 and June 2024. The study was reported in accordance with the Strengthening the Reporting of Observational Studies in Epidemiology (STROBE) guidelines for observational studies. A completed STROBE checklist is provided as Electronic Supplementary Material. Patients with autoimmune rheumatic diseases were recruited at a tertiary rheumatology outpatient clinic of a public university hospital in southeastern Brazil. Control individuals without autoimmune rheumatic diseases were recruited for comparison and selected to achieve close comparability with cases with respect to age, sex, and household income. The final study size was determined by the number of eligible participants with available serum samples and the required demographic, clinical, and laboratory data during the recruitment period; no formal a priori sample size calculation was performed. Patients were classified as having rheumatoid arthritis (RA), systemic lupus erythematosus (SLE), or spondyloarthritis (SpA) according to established international criteria: the 2010 ACR/EULAR classification criteria for RA [29], the 2019 EULAR/ACR classification criteria for SLE [30], and the ASAS classification criteria for SpA [31].

All participants or their legal guardians provided written informed consent prior to enrollment. For participants younger than 18 years, written informed assent was obtained in addition to consent from a parent or legal guardian. This study was approved by the Research Ethics Committee of the Federal University of Uberlândia (CEP/UFU), Brazil, under two research protocols: CAAE 63571422.1.0000.5152, approval number 5.820.607, approved on 15 December 2022; and CAAE 63623822.2.0000.5152, approval number 5.768.585, approved on 21 November 2022.

### Control recruitment and selection

Control participants were recruited from community settings in the same municipality, including schools, educational centers, and residential neighborhoods. Controls were selected at enrollment to achieve close comparability with cases with respect to age, sex, and household income. Eligibility criteria for the control group included self-reported absence of chronic diseases, no previous diagnosis of autoimmune rheumatic disease, and no continuous use of medications for chronic conditions or immunomodulatory therapy.

### Data collection, socioeconomic and clinical assessment

Socioeconomic and environmental information was collected using a structured questionnaire and included household income, pet ownership, self-reported history of helminth infection, anthelmintic use in the previous 6 months, and neighborhood characteristics. To refine socioeconomic characterization, neighborhoods were classified according to the average land price per square meter, using a previously published local classification [32]. Clinical and laboratory data, as well as current pharmacological treatment, including glucocorticoids, disease-modifying antirheumatic drugs (DMARDs), and biologic agents, were obtained from medical records. In patients with SLE, disease activity was assessed using the Systemic Lupus Erythematosus Disease Activity Index (SLEDAI) [33]. Functional disability was assessed using the Health Assessment Questionnaire-Disability Index (HAQ-DI) [34] in patients with RA and SpA. For patients, peripheral blood eosinophil counts were obtained from medical records on the day of blood collection or, when unavailable, from the closest available result within 30 days of sample collection.

### Antigen production and purification

To combine broad serological screening with improved specificity, a sequential antigen strategy was adopted. Native *T. canis* excretory-secretory (TES) antigen was used for initial screening because it represents the conventional serological antigenic preparation for human toxocariasis and contains a broad repertoire of larval secreted antigens. TES-reactive samples were then further evaluated using a recombinant chimeric antigen selected to improve specificity and reduce cross-reactivity. Native *T. canis* excretory-secretory (TES) antigens were produced in vitro as previously described by Thomas et al. (2016) [35]. Briefly, adult female worms were recovered from naturally infected dogs, and eggs were obtained from the uteri of the females. These eggs were incubated in 2% formalin at 28 °C for approximately 35 days until larval embryonation. Embryonated eggs were then processed to obtain larvae, which were maintained in culture for TES collection.

The recombinant chimeric antigen used in this study was developed by a collaborating laboratory and has been described previously [28, 36]. The construct was designed by combining linear B-cell epitopes derived from the *T. canis* TES-26 and CTL-4 proteins, cloned into the pET-21a(+) vector, and expressed in *Escherichia coli* BL21 (DE3). Previous studies reported high diagnostic performance and reduced cross-reactivity with other helminths, supporting its use in the sequential serological strategy adopted in the present study [28, 36].

### Immunological assays

Peripheral blood samples (5 mL) were collected from all participants in tubes without anticoagulant. After centrifugation at 3000 rpm for 10 min, serum aliquots were obtained and stored at − 80 °C until use.

A sequential antigen-based serological strategy was used. All samples were initially screened for anti-*T. canis* IgG using native TES antigen. TES-reactive samples were subsequently tested with the recombinant chimeric antigen for confirmatory IgG assessment and for determination of IgG1 and IgG4 seroreactivity. Importantly, IgG1 and IgG4 assays were performed in all TES-reactive samples, not only in those that were also positive in the confirmatory IgG assay. Final seroreactivity frequencies for recombinant IgG, IgG1, and IgG4 were expressed using the total number of individuals in each study group as the denominator.

Indirect ELISA was performed using 96-well microplates (Corning Costar^®^, Ref. 3590). Wells were coated overnight at 4 °C with 100 µL/well of antigen at optimized concentrations: 3 µg/mL for native TES and 4.5 µg/mL for the recombinant chimeric antigen, diluted in 0.05 M carbonate-bicarbonate buffer (pH 9.6). After washing three times with PBS containing 0.05% Tween-20 (PBS-T), non-specific binding sites were blocked with 5% skim milk (Molico, Nestlé) in PBS-T for 1 h at 37 °C. Serum samples were diluted 1:100 in blocking buffer and incubated for 1 h at 37 °C in duplicate.

For total IgG detection, plates were incubated with peroxidase-conjugated goat anti-human IgG (Fc specific) (Sigma-Aldrich/Merck, Cat. A0170) diluted 1:10,000. For subclass determination, plates were incubated with peroxidase-conjugated mouse monoclonal anti-human IgG1 (clone 8c/6–39, Cat. SAB4200768) diluted 1:4,000, or peroxidase-conjugated mouse monoclonal anti-human IgG4 (clone HP-6025, Cat. SAB4200770) diluted 1:1,000. Reactions were developed with o-phenylenediamine dihydrochloride (OPD) substrate (SIGMAFAST™, P9187, Sigma-Aldrich) for 15 min in the dark and stopped with 50 µL of 2 N H₂SO₄. Absorbance was measured at 490 nm using a microplate reader (BioTek). Negative control sera were run on each plate, and the cut-off was calculated on a plate-by-plate basis as the mean optical density (OD) of the negative controls plus three standard deviations. Results were expressed as reactivity index (RI; sample OD/cut-off OD); samples with RI ≥ 1.0 were considered positive.

IL-10 was assessed in all study groups, whereas IL-17 results are reported descriptively only for patient groups because comparable control data were unavailable; therefore, IL-17 was not used for case-control comparisons. The lowest non-zero standard concentration of the calibration curve was adopted as the lower reporting limit, corresponding to 31.3 pg/mL for IL-10 and 15.6 pg/mL for IL-17. Values below these thresholds were recorded as 0 for descriptive analyses.

### Statistical analysis

Statistical analyses were performed using Python (version 3.10) with the pandas, scipy, statsmodels, and scikit-learn libraries. Continuous variables were compared between groups using the Mann–Whitney U test, whereas categorical variables were compared using Fisher’s exact test. Seroreactivity frequencies were calculated using the total number of individuals in each study group as the denominator. Because controls were selected to achieve comparability with cases at enrollment rather than through individual pair matching, associations between disease status and seroreactivity were estimated using unconditional multivariable logistic regression models adjusted for age, sex, and household income. To account for multiple testing across the nine primary serological models, Benjamini–Hochberg false discovery rate (FDR)-adjusted q values were calculated. Because the IgG4 analysis in SLE involved small cell counts, sensitivity analyses were performed using Fisher’s exact test and Firth penalized logistic regression. Additional sensitivity analyses were performed by extending the main serological models to include pet ownership, neighborhood m² value category, self-reported history of helminth infection, and anthelmintic use in the previous 6 months. Associations between immune markers and clinical outcomes were evaluated using ordinary least squares regression with HC3 robust standard errors for HAQ-DI and negative binomial regression for SLEDAI. In SLE, additional sensitivity analyses evaluated SLEDAI models after adjustment for glucocorticoid, DMARD, and biologic use, and exploratory binary logistic regression models were fitted for SLEDAI > 0 and SLEDAI ≥ 4. These binary SLEDAI sensitivity analyses were considered exploratory because of the low number of events, particularly for SLEDAI ≥ 4. Analyses were performed using complete-case data for each model. For descriptive cytokine analyses, values below the lower reporting limits were recorded as 0, and IL-17 was not formally compared between cases and controls because comparable control data were unavailable or insufficient. All tests were two-tailed. Effect estimates are presented with 95% confidence intervals, while nominal p values and FDR-adjusted q values are reported in tables and online resources, as appropriate.

## Results

### Demographic, socioeconomic, and baseline immunological characteristics

The study included 442 individuals distributed across three disease-specific case-control comparisons. As shown in Table 1, age, sex, and household income were comparable between patients and their respective controls in all three comparisons (all *p* > 0.05). Likewise, history of helminth infection and anthelmintic use within the previous 6 months did not differ significantly between cases and controls in any comparison (all *p* > 0.05). These baseline characteristics provide the descriptive context for the serological analyses presented below, and age, sex, and household income were retained as covariates in adjusted models.

**Table 1:** Demographic, socioeconomic, clinical, cytokine, and serological characteristics of patients with autoimmune rheumatic diseases and their control groups

Variable	RA (*n* = 89)	CT-RA (*n* = 89)	SLE (*n* = 75)	CT-SLE (*n* = 76)	SpA (*n* = 57)	CT-SpA (*n* = 56)	*p*-value (RA vs. CT-RA)	*p*-value (SLE vs. CT-SLE)	*p*-value (SpA vs. CT-SpA)
Demographic characteristics									
Age (years), median [IQR]	51.00 [41.00–59.00]	47.00 [40.00–55.00]	38.00 [28.50–43.50]	39.00 [32.00–47.00]	50.00 [41.00–58.00]	45.50 [31.25–55.25]	0.152	0.231	0.102
Female sex, n (%)	81 (91.0%)	76 (85.4%)	71 (94.7%)	72 (94.7%)	18 (31.6%)	18 (32.1%)	0.353	1.000	1.000
Socioeconomic and environmental variables									
Household income (minimum wages), median [IQR]	2.00 [1.00–3.00]	2.00 [1.00–3.50]	2.00 [1.50–3.00]	2.00 [1.38–3.00]	2.00 [1.50–2.50]	2.00 [1.00–4.12]	0.110	0.666	0.548
Neighborhood m² value category, median [IQR]	2.00 [2.00–4.00]	4.00 [3.00–5.00]	4.00 [2.25–4.00]	4.00 [2.00–5.00]	3.00 [2.00–4.50]	3.00 [2.00–4.00]	< 0.001	0.245	0.596
Pet ownership (dog/cat), n (%)	57 (64.0%)	63 (70.8%)	56 (74.7%)	55 (72.4%)	47 (82.5%)	28 (50.0%)	0.424	0.854	< 0.001
History of helminth infection, n (%)	38 (42.7%)	30 (33.7%)	28 (37.3%)	33 (43.4%)	23 (40.4%)	16 (28.6%)	0.280	0.508	0.236
Anthelmintic use in the last 6 months, n (%)	11 (12.4%)	16 (18.0%)	24 (32.0%)	17 (22.4%)	8 (14.0%)	8 (14.3%)	0.404	0.204	1.000
Cytokines and eosinophils									
IL-10 (pg/mL), median [IQR]	0.00 [0.00–0.00]	0.00 [0.00–0.00]	0.00 [0.00–0.00]	0.00 [0.00–0.00]	0.00 [0.00–0.00]	0.00 [0.00–0.00]	0.061	0.300	0.040
IL-10 detectable, n (%)	10 (11.2%)	18 (20.2%)	3 (4.0%)	1 (1.3%)	3 (5.3%)	10 (17.9%)	0.149	0.367	0.043
IL-17 (pg/mL), median [IQR]	0.00 [0.00–0.00]	–	0.00 [0.00–0.00]	–	0.00 [0.00–0.00]	–	–	–	–
IL-17 detectable, n (%)	6 (6.7%)	–	6 (8.0%)	–	1 (1.8%)	–	–	–	–
Eosinophil count (/mm³), median [IQR]	161.00 [82.00–249.00]	–	78.00 [33.00–127.00]	–	161.50 [111.25–258.00]	–	–	–	–
Clinical indices									
HAQ-DI, median [IQR]	1.00 [0.38–1.62]	–	–	–	1.00 [0.25–1.38]	–	–	–	–
SLEDAI score, median [IQR]	–	–	0.00 [0.00–2.00]	–	–	–	–	–	–
Serological reactivity to the recombinant chimeric antigen									
IgG reactive, n (%)	33 (37.1%)	22 (24.7%)	28 (37.3%)	16 (21.1%)	26 (45.6%)	18 (32.1%)	0.104	0.032	0.178
IgG1 reactive, n (%)	10 (11.2%)	11 (12.4%)	5 (6.7%)	11 (14.5%)	11 (19.3%)	7 (12.5%)	1.000	0.185	0.442
IgG4 reactive, n (%)	10 (11.2%)	7 (7.9%)	2 (2.7%)	9 (11.8%)	9 (15.8%)	4 (7.1%)	0.611	0.056	0.238

Data are presented as median [IQR] for continuous variables and n (%) for categorical variables, unless otherwise indicated. P values compare each disease group with its respective control group. Percentages were calculated using the fixed total sample size of each group. — indicates not available or not assessed in a comparable manner. IL-10 and IL-17 values below the lower reporting limit were recorded as 0 for descriptive analysis. IL-17 results are reported only for patient groups because comparable control data were unavailable; therefore, control groups are shown as —. For serology, all participants were initially screened by TES-IgG, and TES-reactive samples were subsequently tested for confirmatory IgG, IgG1, and IgG4 assays using the recombinant chimeric antigen; however, the frequencies of IgG, IgG1, and IgG4 reactivity are expressed in relation to the total number of individuals in each group. P values were obtained using Mann–Whitney U tests for continuous variables and Fisher’s exact tests for categorical variables

*RA* rheumatoid arthritis, *SLE* systemic lupus erythematosus, *SpA* spondyloarthritis, *CT* corresponding control group, *HAQ-DI* Health Assessment Questionnaire-Disability Index, *SLEDAI* Systemic Lupus Erythematosus Disease Activity Index

Differences in other baseline variables were disease-specific. In the RA comparison, the neighborhood m² value category was lower among patients than controls (2.00 [2.00–4.00] vs. 4.00 [3.00–5.00], *p* < 0.001). In the SpA comparison, pet ownership was more frequent among patients than controls (82.5% vs. 50.0%, *p* < 0.001). No significant differences in these variables were observed in the SLE versus CT-SLE comparison.

Regarding cytokine-related variables, IL-10 did not differ significantly between RA and CT-RA or between SLE and CT-SLE. By contrast, SpA showed nominally lower IL-10 levels than CT-SpA, both in median values (*p* = 0.040) and in the proportion of detectable samples (*p* = 0.043). IL-17 results are reported descriptively only for patient groups because comparable control data were unavailable. Therefore, IL-17 was not directly compared between cases and controls. Eosinophil counts were also available only for case groups and are presented descriptively in Table 1. Detailed cytokine detectability and case-control comparisons are provided in Online Resource 5.

### Disease-specific patterns of anti-*T. canis* seroreactivity

**Fig. 1:**
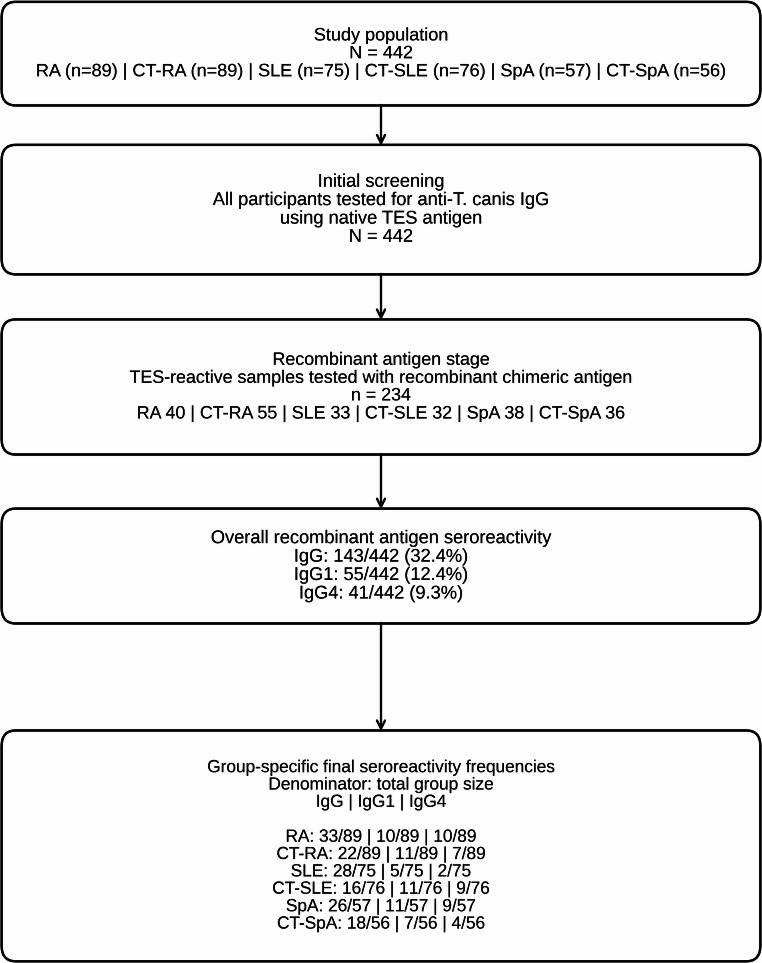
Sequential serological testing strategy and final group-specific denominators. Legend: All participants were initially screened for anti-*Toxocara canis* IgG using native TES antigen. TES-reactive samples were subsequently evaluated using the recombinant chimeric antigen for IgG, IgG1, and IgG4 seroreactivity. Final seroreactivity frequencies were expressed using the total number of individuals in each disease-specific group as the denominator. *TES Toxocara* excretory-secretory antigen, *RA* rheumatoid arthritis, *SLE* systemic lupus erythematosus, *SpA* spondyloarthritis, *CT* corresponding control group, *IgG* immunoglobulin G

**Fig. 2:**
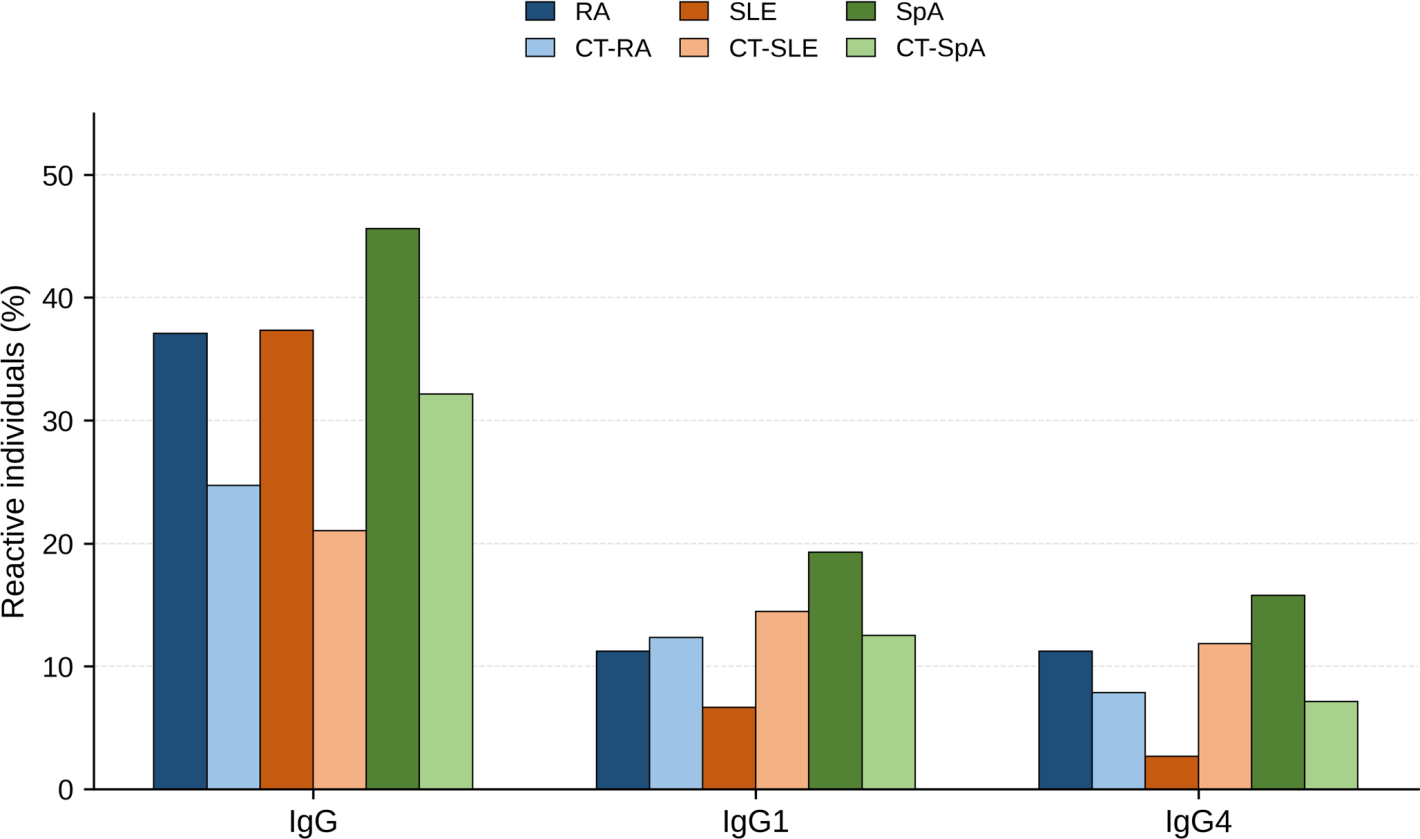
Anti-*Toxocara canis* seroreactivity across autoimmune rheumatic disease groups and controls. Legend: Bars show the observed percentage of individuals reactive for IgG, IgG1, and IgG4 in each disease-specific case-control comparison. Frequencies for IgG1 and IgG4 were expressed using the total number of individuals in each group as the denominator, although subclass testing was performed among TES-reactive samples. No error bars are shown because the figure presents observed proportions descriptively. Inferential statistics from adjusted logistic regression models, including nominal p values and FDR-adjusted q values, are provided in Table 2. *RA* rheumatoid arthritis, *SLE* systemic lupus erythematosus, *SpA* spondyloarthritis, *CT* corresponding control group, *IgG* immunoglobulin G, *TES Toxocara* excretory-secretory antigen, *FDR* false discovery rate

The sequential serological testing strategy and group-specific denominators are summarized in Fig. 1. Analysis of recombinant antigen seroreactivity showed that anti-*T. canis* IgG antibodies were frequently detected across the study population (Fig. 2). In adjusted logistic regression models, SLE was the only disease group showing a nominally significant association with IgG seroreactivity compared with its respective control group (OR 2.44, 95% CI 1.16–5.14) (Table 2). RA and SpA also showed higher adjusted point estimates for IgG seroreactivity than their respective control groups, although the corresponding confidence intervals crossed the null value (RA: OR 1.90, 95% CI 0.98–3.68; SpA: OR 2.26, 95% CI 0.99–5.19) (Table 2). Nominal p values and FDR-adjusted q values are provided in Table 2. However, no association remained statistically significant after FDR correction. These findings suggest a directionally consistent tendency toward higher total anti-*T. canis* IgG seroreactivity across autoimmune rheumatic diseases, with the strongest nominal evidence observed in SLE.

**Table 2:** Adjusted associations between anti-*Toxocara* seroreactivity and autoimmune disease status

Comparison	Marker	Case reactive, *n*/*N* (%)	Control reactive, *n*/*N* (%)	Adjusted OR (95% CI)	Nominal *p* value	FDR-adjusted q value	*N* included
RA vs. CT-RA	IgG	33/89 (37.1%)	22/89 (24.7%)	1.90 (0.98–3.68)	0.056	0.127	178
RA vs. CT-RA	IgG1	10/89 (11.2%)	11/89 (12.4%)	0.82 (0.33–2.08)	0.683	0.683	178
RA vs. CT-RA	IgG4	10/89 (11.2%)	7/89 (7.9%)	1.71 (0.59–4.91)	0.322	0.414	178
SLE vs. CT-SLE	IgG	28/75 (37.3%)	16/76 (21.1%)	2.44 (1.16–5.14)	0.019	0.127	151
SLE vs. CT-SLE	IgG1	5/75 (6.7%)	11/76 (14.5%)	0.40 (0.13–1.24)	0.112	0.167	151
SLE vs. CT-SLE	IgG4	2/75 (2.7%)	9/76 (11.8%)	0.20 (0.04–0.99)	0.049	0.127	151
SpA vs. CT-SpA	IgG	26/57 (45.6%)	18/56 (32.1%)	2.26 (0.99–5.19)	0.054	0.127	113
SpA vs. CT-SpA	IgG1	11/57 (19.3%)	7/56 (12.5%)	1.46 (0.49–4.31)	0.493	0.555	113
SpA vs. CT-SpA	IgG4	9/57 (15.8%)	4/56 (7.1%)	3.30 (0.88–12.42)	0.078	0.140	113

Multivariable logistic regression models comparing rheumatoid arthritis (RA), systemic lupus erythematosus (SLE), and spondyloarthritis (SpA) groups with their corresponding control groups (CT-RA, CT-SLE, and CT-SpA). Models were adjusted for age, sex, and household income. Seroreactivity frequencies are presented as n/N (%) for each case and control group. Adjusted odds ratios (ORs) with 95% confidence intervals (95% CIs), nominal p values, and Benjamini-Hochberg false discovery rate (FDR)-adjusted q values are shown. FDR correction was applied across the nine primary serological models. No association remained statistically significant after FDR correction

The most distinct subclass pattern was observed for IgG4 in SLE. SLE showed lower odds of IgG4 seroreactivity than CT-SLE in the standard adjusted logistic regression model (OR 0.20, 95% CI 0.04–0.99) (Table 2), whereas IgG4 differences in RA and SpA were not nominally significant. Because this comparison involved a small number of IgG4-reactive individuals in SLE, additional small-cell sensitivity analyses were performed. The direction of the association remained consistent in the crude Fisher exact test and in Firth penalized logistic regression, but these analyses did not reach conventional statistical significance (Online Resource 2).

Sensitivity analyses additionally adjusted for pet ownership, neighborhood m² value category, self-reported history of helminth infection, and anthelmintic use in the previous 6 months are presented in Online Resource 3. In these models, the association between SLE and total IgG seroreactivity remained nominally significant, whereas the IgG4 association in SLE retained the same direction but was no longer statistically significant. Associations for total IgG in RA and SpA were attenuated in the extended models.

### Clinical correlates of immune markers

Associations between immune markers and clinical outcomes were evaluated using disease-specific models. In RA and SpA, no independent association was detected between seroreactivity markers and HAQ-DI after adjustment. In both groups, household income was inversely associated with HAQ-DI (Online Resource 1).

**Fig. 3:**
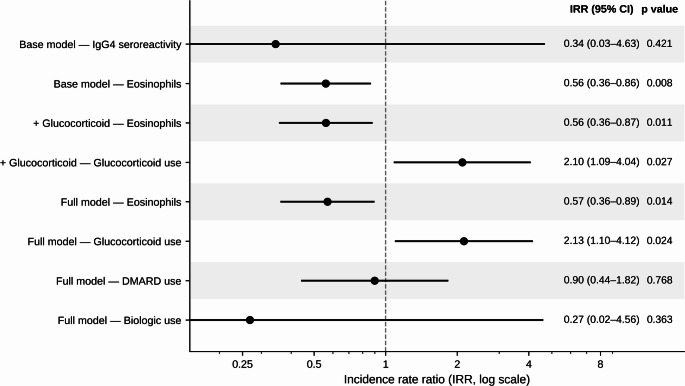
Sensitivity analyses of eosinophil-associated SLEDAI variation in systemic lupus erythematosus. Legend: Forest plot showing incidence rate ratios (IRRs) and 95% confidence intervals from negative binomial regression models evaluating SLEDAI as a count outcome in patients with systemic lupus erythematosus. Eosinophil count was modeled per 100 cells/mm³. The association between higher eosinophil counts and lower SLEDAI scores persisted after adjustment for glucocorticoid use and after additional adjustment for DMARD and biologic use. Horizontal lines represent 95% confidence intervals. *SLEDAI* Systemic Lupus Erythematosus Disease Activity Index, *SLE* systemic lupus erythematosus, *DMARD* disease-modifying antirheumatic drug, *IRR* incidence rate ratio, *CI* confidence interval. These analyses should be interpreted as exploratory because SLEDAI values were low overall and the number of patients with clinically active disease was limited

In SLE, disease activity was modeled using negative binomial regression. SLEDAI scores were low overall, with a median of 0 [0–2], and 42/75 patients (56.0%) had SLEDAI = 0 (Online Resource 4). Higher eosinophil counts were associated with lower SLEDAI scores in the base model (IRR per 100 eosinophils/mm³: 0.56, 95% CI 0.36–0.86), and this association persisted after adjustment for glucocorticoid use and after additional adjustment for DMARD and biologic use (Fig. 3; Online Resource 4). These findings should be interpreted as exploratory because SLEDAI scores were low overall and the number of patients with clinically active disease was limited. IgG4 seroreactivity was not independently associated with SLEDAI. No detectable differences in glucocorticoid, DMARD, or biologic use were observed according to IgG4 seroreactivity in SLE; however, this comparison was constrained by the extremely small number of IgG4-reactive individuals (Online Resource 4).

## Discussion

Our findings show that anti-*Toxocara* seroreactivity was frequent across autoimmune rheumatic diseases and suggest disease-specific differences in the qualitative profile of this response. For total IgG, all three disease groups showed higher adjusted point estimates than their corresponding control groups, although nominal statistical significance was observed only in SLE and no association remained significant after FDR correction. The most distinct subclass pattern was also observed in SLE, where higher total IgG seroreactivity coexisted with a nominal signal of lower IgG4 seroreactivity. Because the IgG4 result was close to the conventional significance threshold, did not remain significant after FDR correction or in small-cell sensitivity analyses, and involved few IgG4-reactive individuals, it should be interpreted as a cautious, hypothesis-generating signal rather than definitive evidence of IgG4 suppression. The use of a recombinant chimeric antigen designed to improve specificity and reduce cross-reactivity relative to native antigens supports the interpretation that these findings may reflect differences in the quality of the anti-*T. canis* humoral response rather than merely nonspecific cross-reactivity [11].

This interpretation should be considered in light of the sequential serological strategy adopted in the present study. All samples were initially screened for IgG using native TES antigen, whereas TES-reactive samples were subsequently evaluated with the recombinant chimeric antigen for confirmatory IgG assessment and subclass determination. Accordingly, the coexistence of higher total IgG seroreactivity and a nominal signal of lower IgG4 seroreactivity in SLE does not represent an internal inconsistency, but rather suggests that broad serological reactivity to *Toxocara* antigens may be maintained or enhanced, while the IgG4 component of this response may be relatively less frequent.

This pattern is noteworthy because SLE is classically associated with B-cell hyperactivity and broad antibody production [27]. In that context, the nominally lower IgG4 seroreactivity observed in SLE does not suggest a generalized inability to mount humoral responses, but rather raises the possibility of a qualitative shift in the anti-helminth response. In chronic human helminth infections, IgG4 has been linked to more regulated immune profiles [19], and more recent work has reinforced the concept of IgG4 as a tolerance-associated isotype whose induction is closely linked to IL-10 and chronic antigen exposure [37]. Experimental and review data in lupus also suggest that helminth-derived immunomodulation may attenuate disease through regulatory mechanisms [23, 38]. Taken together, our data are compatible with the possibility that the regulatory-associated humoral features of anti-*Toxocara* immunity may differ in SLE, although this interpretation should remain cautious because the IgG4 finding was exploratory, did not remain significant after FDR correction or in small-cell sensitivity analyses, and involved few IgG4-reactive individuals.

At the clinical level, the most consistent disease-specific correlate identified in the present study was eosinophil count in SLE. Higher eosinophil counts were associated with lower SLEDAI scores, and this association persisted after adjustment for glucocorticoid use and after additional adjustment for DMARD and biologic use, whereas IgG4 seroreactivity was not independently associated with disease activity in the within-group model. However, this association should be interpreted as exploratory because SLEDAI values were low overall and the number of patients with clinically active disease was limited. This distinction is important because the between-group comparison suggested a nominal signal of lower IgG4 seroreactivity in SLE relative to the corresponding control group, whereas the independent clinical correlate detected within SLE was eosinophil count rather than IgG4 itself. In helminth infections, eosinophils may accompany distinct immunological phases. Early or more inflammatory stages may be characterized by eosinophil-rich type 2 responses that can be granulomagenic and pathogenic, as described in experimental toxocariasis [16, 17], whereas chronic infection may evolve toward a more modulated type 2/regulatory profile [39]. In this context, our findings should not be interpreted as evidence that eosinophils are directly protective in SLE. Rather, eosinophil counts may represent an indirect marker of a broader immune context associated with lower disease activity, potentially influenced by type 2-biased immunity, previous helminth exposure, treatment patterns, or other unmeasured factors. This cautious interpretation is compatible with recent clinical observations linking peripheral eosinophil counts to disease activity and outcomes in lupus nephritis [40].

The results in RA and SpA should be interpreted cautiously, but they may still be informative as exploratory directional patterns. For total IgG seroreactivity, both RA and SpA showed higher adjusted point estimates than their corresponding control groups, but the evidence was not robust; these associations did not remain significant after FDR correction and were attenuated in the extended sensitivity models. These findings suggest that higher anti-*Toxocara* IgG seroreactivity may not be exclusive to SLE, although the statistical support was strongest and most consistent in SLE. Helminth-derived molecules have been reported to attenuate inflammatory pathways in experimental arthritis models [26, 41], and we therefore considered the possibility that anti-*Toxocara* serological markers might correlate with functional status in human autoimmune rheumatic diseases. However, in RA and SpA, no independent association was detected between IgG, IgG1, or IgG4 seroreactivity and HAQ-DI after adjustment. In both groups, household income remained inversely associated with HAQ-DI, whereas the serological markers did not. This finding is consistent with previous evidence linking lower socioeconomic status to worse functional outcomes in rheumatoid arthritis and spondyloarthritis, potentially reflecting differences in access to care, treatment continuity, comorbidity burden, and broader social determinants of health [42, 43]. These findings suggest that the relationship between helminth-related serological markers and clinical expression may vary across autoimmune rheumatic diseases and may be outweighed by social determinants of functional disability in RA and SpA.

The cytokine findings also require cautious interpretation. Between-group differences in IL-10 were not observed for SLE versus its corresponding control group, whereas nominally lower IL-10 values and lower IL-10 detectability were observed in SpA relative to CT-SpA. Because serum IL-10 values were strongly concentrated at the lower end of the assay range, these findings should be interpreted conservatively. IL-17 results were reported descriptively only for patient groups because comparable control data were unavailable, and IL-17 detectability was low. Therefore, the cytokine results should not be interpreted as robust mechanistic evidence. More broadly, although SLE has often been discussed in the context of Th17/Treg disequilibrium and impaired regulatory pathways [44, 45], our serum cytokine results do not support a simple model in which SLE is uniquely defined by lower circulating IL-10 than controls. Rather, they suggest that serum IL-10 may not be a sufficiently stable standalone surrogate of regulatory status in this setting.

The higher adjusted point estimates for total anti-*Toxocara* IgG seroreactivity observed across the three disease groups also warrant consideration. Because anti-*Toxocara* serology reflects exposure and humoral recognition rather than active infection itself, these findings cannot be interpreted as evidence of ongoing toxocariasis [10–12]. Several non-mutually exclusive explanations may be considered, including differential cumulative exposure, persistence of antibody responses after previous exposure, and disease-related differences in humoral responsiveness. This latter explanation may be particularly relevant in SLE, in which B-cell hyperactivity and broad antibody production provide a biological context for higher total IgG seroreactivity [27]. In RA and SpA, the higher point estimates followed the same direction but were not nominally significant, did not remain significant after FDR correction, and were attenuated in the extended sensitivity models; they should therefore be interpreted as exploratory signals rather than definitive disease-specific associations.

From an epidemiological perspective, the recruitment strategy based on comparability between cases and controls supports the interpretation of the serological findings. Patients and controls were comparable with respect to age, sex, and household income, and no significant between-group differences were observed for reported history of helminth infection or recent anthelmintic use. This reduces the likelihood that the main serological differences were driven primarily by major demographic or socioeconomic imbalance. Notably, the disease-specific baseline differences observed in neighborhood m² value category in RA and pet ownership in SpA did not parallel the main serological pattern, which was most consistently observed in SLE. Although pet ownership is epidemiologically relevant to *Toxocara* exposure, its higher frequency in SpA was not accompanied by a statistically significant adjusted serological association and was further considered in extended sensitivity analyses, indicating that this variable alone is unlikely to explain the main findings. At the same time, the frequent anti-*Toxocara* seroreactivity observed across groups is compatible with substantial cumulative exposure in this setting, which is consistent with the known epidemiology of toxocariasis in Brazil and with the public health relevance of canine and feline environmental contamination in urban areas [9, 12–14, 46].

The possible influence of treatment must also be interpreted with caution. Immunosuppressive therapy could theoretically affect susceptibility to infection, persistence of antigenic stimulation, eosinophil counts, or antibody profiles. However, the present data do not allow these possibilities to be established. No detectable differences in glucocorticoid, DMARD, or biologic use were observed according to IgG4 seroreactivity in SLE, although this comparison was constrained by the extremely small number of IgG4-reactive individuals. In addition, the inverse association between eosinophil counts and SLEDAI persisted after adjustment for glucocorticoid use and after additional adjustment for DMARD and biologic use. Therefore, although treatment effects cannot be excluded, the available analyses do not support the interpretation that the nominally lower IgG4 seroreactivity in SLE or the eosinophil–SLEDAI association was simply explained by immunosuppressive therapy.

These findings should be interpreted in light of several methodological considerations. The cross-sectional design precludes causal inference regarding helminth exposure, anti-*Toxocara* immune profiles, and autoimmune disease trajectories. In addition, subclass analyses were restricted to TES-reactive samples, reflecting the sequential serological strategy adopted in this study. This design allowed initial screening with the conventional TES antigen, followed by more specific characterization of TES-reactive samples using a recombinant chimeric antigen, but it should be considered when interpreting the relationship between total IgG and subclass frequencies. Although this strategy was intended to improve the specificity of downstream serological characterization, serology remains an indirect marker of exposure and humoral recognition rather than definitive evidence of active infection. Some subgroup analyses were also constrained by small numbers, particularly the low frequency of IgG4-reactive individuals in SLE. Similarly, the low overall SLEDAI distribution and the limited number of patients with clinically active disease constrain the interpretation of eosinophil–SLEDAI models. Accordingly, nominal or borderline findings, especially those involving IgG4 in SLE and total IgG in RA and SpA, should be interpreted as hypothesis-generating and require confirmation in larger cohorts. Additional limitations include the self-reported absence of chronic diseases and medication use among controls, the lack of eosinophil counts in control groups, and the low detectability of serum cytokines, particularly for IL-17 comparisons. These considerations should be balanced against important strengths, including the use of control groups selected to achieve comparability with cases for major demographic and socioeconomic variables, the sequential use of TES and recombinant antigens, and the integration of serological, exploratory cytokine, patient eosinophil, and clinical data across three autoimmune rheumatic disease groups.

In summary, our findings suggest that autoimmune rheumatic diseases may not share a uniform anti-*Toxocara* immune profile. Total IgG seroreactivity showed higher adjusted point estimates across disease groups, although nominal statistical significance was observed only in SLE and no association remained significant after FDR correction. The most distinctive pattern was observed in SLE, where higher total IgG seroreactivity coexisted with a cautious, hypothesis-generating signal of lower IgG4 seroreactivity, and where eosinophil counts showed an exploratory independent association with lower SLEDAI scores. Together, these findings suggest that the quality, rather than merely the presence, of anti-helminth humoral immunity may differ across autoimmune disease contexts. They also reinforce the view that helminth-related immune signatures are context-dependent rather than uniformly protective across autoimmune settings [25, 47].

## Supplementary Information

Below is the link to the electronic supplementary material.

Supplementary Material 1

## Declarations

### AI-use statement

No AI tool was used to generate study data, perform statistical analyses, create scientific figures or tables, select references, interpret results, or formulate the scientific conclusions of this report. AI-assisted tools, including OpenAI ChatGPT, were used only for limited editorial support of author-written text. All content was critically reviewed, verified, and approved by the authors.

### Previous presentations

Preliminary and partial results related to this study were presented at scientific congresses: (1) Rabello LC, Miranda VC, Scucuglia BM, Paula TS, Resende HM, Pinheiro NB, Morais CG, Silva RC, Pinheiro CS, Cambraia-Miranda RR. Differential IgG seroreactivity to *Toxocara canis* in immunocompetent and autoimmune rheumatic patients: frequency, associations, and functional correlations. Oral presentation at the 29th Brazilian Congress of Parasitology – PARASITO 2025; 22–25 April 2025; Salvador, Bahia, Brazil. DOI: not available. (2) Rabello LC, Miranda VC, Paula TS, Ferreira IA, Lima LVO, Scucuglia BM, Resende HM, Ranza R, Pinheiro NB, Silva RC, Morais CG, Pinheiro CS, Cambraia-Miranda RR. Avaliação da resposta IgG, IgG1 e IgG4 a antígenos de *Toxocara canis* em indivíduos com artrite reumatoide e controles pareados. E-poster presentation, abstract no. 9175, 60th Congress of the Brazilian Society of Tropical Medicine – MEDTROP 2025; 2–5 November 2025; João Pessoa, Paraíba, Brazil. DOI: not available. These congress communications reported preliminary or partial analyses and do not constitute duplicate publication of the present manuscript.

### Commercial editing

No external commercial editing agency or agent was used in the preparation or revision of this manuscript.
