# Supplementary material for: Anti-Toxocara canis seroreactivity across autoimmune rheumatic diseases with exploratory IgG4 and eosinophil-associated signals in systemic lupus erythematosus: a cross-sectional case-control study
Source: Rheumatol Int. 2026 Jul 16;46(8):212. doi: 10.1007/s00296-026-06249-3 (PMC13375815; doi:10.1007/s00296-026-06249-3)
Supplement: Supplementary file 1 — Supplementary Material 1 [file 296_2026_6249_MOESM1_ESM.docx]

**Online Resource 1. Multivariable linear regression models evaluating predictors of functional disability (HAQ-DI) in patients with rheumatoid arthritis and spondyloarthritis**

| **Group** | **Variable** | **N included** | **β (95% CI)** | **P value** | **R²** |
| --- | --- | --- | --- | --- | --- |
| RA | Intercept | 85 | 1.361 (0.507–2.216) | 0.002 | 0.216 |
| RA | IgG4 reactive (vs negative) | 85 | -0.262 (-0.740–0.216) | 0.282 | 0.216 |
| RA | Eosinophil count (/mm³) | 85 | -0.001 (-0.001–0.000) | 0.168 | 0.216 |
| RA | Household income (minimum wages) | 85 | -0.217 (-0.325 – -0.109) | < 0.001 | 0.216 |
| RA | Age (years) | 85 | 0.007 (-0.007–0.021) | 0.347 | 0.216 |
| RA | Male sex (vs female) | 85 | -0.139 (-0.779–0.501) | 0.670 | 0.216 |
| SpA | Intercept | 48 | 1.552 (0.589–2.516) | 0.002 | 0.275 |
| SpA | IgG4 reactive (vs negative) | 48 | -0.351 (-0.997–0.295) | 0.287 | 0.275 |
| SpA | Eosinophil count (/mm³) | 48 | 0.000 (-0.001–0.002) | 0.429 | 0.275 |
| SpA | Household income (minimum wages) | 48 | -0.254 (-0.379 – -0.130) | < 0.001 | 0.275 |
| SpA | Age (years) | 48 | 0.001 (-0.014–0.017) | 0.871 | 0.275 |
| SpA | Male sex (vs female) | 48 | -0.133 (-0.491–0.224) | 0.465 | 0.275 |

*Notes: Ordinary least squares linear regression models with HC3 robust standard errors evaluating predictors of functional disability, measured by the Health Assessment Questionnaire-Disability Index (HAQ-DI), in patients with rheumatoid arthritis (RA) and spondyloarthritis (SpA). Models included IgG4 seropositivity, eosinophil count, household income, age, and sex. Regression coefficients (β), 95% confidence intervals (95% CIs), p values, and model R² are shown. Negative coefficients indicate association with lower HAQ-DI scores.*

**Online Resource 2. Small-cell sensitivity analyses for IgG4 seroreactivity in systemic lupus erythematosus**

| **Analysis** | **Comparison** | **Adjustment** | **SLE reactive/total** | **CT-SLE reactive/total** | **OR (95% CI)** | **p value** | **N included** |
| --- | --- | --- | --- | --- | --- | --- | --- |
| Crude Fisher exact test | SLE vs CT-SLE | None | 2/75 | 9/76 | 0.20 | 0.056 | 151 |
| Standard logistic regression | SLE vs CT-SLE | Age, sex, household income | 2/75 | 9/76 | 0.20 (0.04–0.99) | 0.049 | 151 |
| Firth penalized logistic regression | SLE vs CT-SLE | Age, sex, household income | 2/75 | 9/76 | 0.25 (0.05–1.11) | 0.068 | 151 |

*Notes: Sensitivity analyses evaluating the association between systemic lupus erythematosus (SLE) and IgG4 seroreactivity compared with the corresponding control group (CT-SLE). The crude comparison was assessed using Fisher’s exact test; for this analysis, the odds ratio from the 2×2 table is shown without a confidence interval. Standard logistic regression and Firth penalized logistic regression models were adjusted for age, sex, and household income. Firth penalized logistic regression was performed because the number of IgG4-reactive individuals in the SLE group was small. The direction of the association remained consistent across analyses, but the Fisher exact test and the Firth model did not reach conventional statistical significance; therefore, this finding should be interpreted as exploratory.*

**Online Resource 3. Sensitivity analyses additionally adjusted for environmental and exposure-related covariates**

| **Comparison** | **Marker** | **Main model  OR (95% CI)** | **Main model  p value** | **Main model N** | **Extended model  OR (95% CI)** | **Extended model p value** | **Extended model N** |
| --- | --- | --- | --- | --- | --- | --- | --- |
| RA vs CT-RA | IgG | 1.90 (0.98–3.68) | 0.056 | 178 | 1.18 (0.53–2.60) | 0.688 | 142 |
| RA vs CT-RA | IgG1 | 0.82 (0.33–2.08) | 0.683 | 178 | 0.39 (0.11–1.34) | 0.134 | 142 |
| RA vs CT-RA | IgG4 | 1.71 (0.59–4.91) | 0.322 | 178 | 1.76 (0.50–6.21) | 0.379 | 142 |
| SLE vs CT-SLE | IgG | 2.44 (1.16–5.14) | 0.019 | 151 | 3.17 (1.29–7.75) | 0.012 | 112 |
| SLE vs CT-SLE | IgG1 | 0.40 (0.13–1.24) | 0.112 | 151 | 0.45 (0.12–1.69) | 0.234 | 112 |
| SLE vs CT-SLE | IgG4 | 0.20 (0.04–0.99) | 0.049 | 151 | 0.29 (0.06–1.47) | 0.135 | 112 |
| SpA vs CT-SpA | IgG | 2.26 (0.99–5.19) | 0.054 | 113 | 1.58 (0.60–4.15) | 0.353 | 91 |
| SpA vs CT-SpA | IgG1 | 1.46 (0.49–4.31) | 0.493 | 113 | 1.18 (0.32–4.37) | 0.802 | 91 |
| SpA vs CT-SpA | IgG4 | 3.30 (0.88–12.42) | 0.078 | 113 | 2.81 (0.59–13.41) | 0.194 | 91 |

*Note: Sensitivity analyses comparing the main logistic regression models with extended models additionally adjusted for pet ownership, neighborhood m² value category, self-reported history of helminth infection, and anthelmintic use in the previous 6 months. Main models were adjusted for age, sex, and household income. Extended models were fitted using complete-case data for all included covariates, resulting in lower sample sizes than the main models. Odds ratios (ORs) and 95% confidence intervals (95% CIs) refer to the association between autoimmune rheumatic disease status and each seroreactivity marker within each disease-specific case-control comparison. RA, rheumatoid arthritis; SLE, systemic lupus erythematosus; SpA, spondyloarthritis; CT, corresponding control group.*

**Online Resource 4. Medication, eosinophil, and SLEDAI sensitivity analyses in systemic lupus erythematosus**

| **Section** | **Analysis / variable** | **Result** | **p value** | **N** |
| --- | --- | --- | --- | --- |
| A. SLEDAI distribution | SLEDAI, median [IQR] | 0 [0–2] | — | 75 |
|  | SLEDAI maximum | 15 | — | 75 |
|  | SLEDAI = 0, n (%) | 42 (56.0%) | — | 75 |
|  | SLEDAI > 0, n (%) | 33 (44.0%) | — | 75 |
|  | SLEDAI ≥ 4, n (%) | 14 (18.7%) | — | 75 |
| B. IgG4 seroreactivity according to medication use | Glucocorticoid use | 1/40 exposed vs 1/35 unexposed IgG4-reactive | 1.000 | 75 |
|  | DMARD use | 2/50 exposed vs 0/25 unexposed IgG4-reactive | 0.550 | 75 |
|  | Biologic use | 0/5 exposed vs 2/70 unexposed IgG4-reactive | 1.000 | 75 |
| C. Eosinophil counts according to medication use | Glucocorticoid use | Exposed vs unexposed: 81.0 [34.2–124.5] vs 72.0 [32.5–137.0] cells/mm³ | 0.880 | 69 |
|  | DMARD use | Exposed vs unexposed: 88.0 [34.8–127.5] vs 61.0 [26.0–107.0] cells/mm³ | 0.424 | 69 |
|  | Biologic use | Exposed vs unexposed: 89.0 [60.0–145.5] vs 77.0 [33.2–126.8] cells/mm³ | 0.712 | 69 |
| D. Negative binomial models for SLEDAI | Eosinophils, base model | IRR 0.56 (0.36–0.86) | 0.008 | 69 |
|  | Eosinophils, + glucocorticoid | IRR 0.56 (0.36–0.87) | 0.011 | 69 |
|  | Eosinophils, + glucocorticoid + DMARD + biologic | IRR 0.57 (0.36–0.89) | 0.014 | 69 |
|  | Glucocorticoid use, adjusted model | IRR 2.10 (1.09–4.04) | 0.027 | 69 |
|  | Glucocorticoid use, fully adjusted model | IRR 2.13 (1.10–4.12) | 0.024 | 69 |
| E. Binary SLEDAI sensitivity models | Eosinophils, SLEDAI > 0, base model | OR 0.64 (0.35–1.16) | 0.141 | 69 |
|  | Eosinophils, SLEDAI > 0, fully adjusted model | OR 0.65 (0.36–1.19) | 0.162 | 69 |
|  | Eosinophils, SLEDAI ≥ 4, + glucocorticoid | OR 0.21 (0.05–0.83) | 0.026 | 69 |
|  | Eosinophils, SLEDAI ≥ 4, fully adjusted model | OR 0.25 (0.06–1.00) | 0.051 | 69 |

*Notes: This online resource summarizes sensitivity analyses in the systemic lupus erythematosus (SLE) subgroup. SLEDAI distribution is shown because disease activity scores were low and contained many zero values. In section B, IgG4 seroreactivity according to medication exposure is presented as IgG4-reactive individuals among exposed versus unexposed patients and was compared using Fisher’s exact test. In section C, eosinophil counts according to medication exposure are shown as median [IQR] cells/mm³ among exposed versus unexposed patients and were compared using Mann–Whitney U tests. Negative binomial regression models evaluated SLEDAI as a count outcome; incidence rate ratios (IRRs) are shown per 100 eosinophils/mm³ for eosinophil count. Binary logistic regression models for SLEDAI > 0 and SLEDAI ≥ 4 were used as exploratory sensitivity analyses. Binary models for SLEDAI ≥ 4 should be interpreted cautiously because of low event numbers and evidence of model instability. The combined biologic + DMARD exposure category is not shown in the main table because only one patient was exposed. SLEDAI, Systemic Lupus Erythematosus Disease Activity Index; DMARD, disease-modifying antirheumatic drug; OR, odds ratio; IRR, incidence rate ratio; CI, confidence interval*.

**Online Resource 5. Serum cytokine detectability and case-control comparisons**

| **Section** | **Cytokine** | **Group / comparison** | **Median [IQR]** | **Detectable, n/N (%)** | **Continuous p value** | **Detectability p value** | **Status / interpretation** |
| --- | --- | --- | --- | --- | --- | --- | --- |
| A. IL-10 detectability by group | IL-10 | RA | 0.00 [0.00–0.00] | 10/89 (11.2%) | — | — | Most values were below the reporting limit |
|  | IL-10 | CT-RA | 0.00 [0.00–0.00] | 18/89 (20.2%) | — | — | Most values were below the reporting limit |
|  | IL-10 | SLE | 0.00 [0.00–0.00] | 3/75 (4.0%) | — | — | Most values were below the reporting limit |
|  | IL-10 | CT-SLE | 0.00 [0.00–0.00] | 1/76 (1.3%) | — | — | Most values were below the reporting limit |
|  | IL-10 | SpA | 0.00 [0.00–0.00] | 3/57 (5.3%) | — | — | Most values were below the reporting limit |
|  | IL-10 | CT-SpA | 0.00 [0.00–0.00] | 10/56 (17.9%) | — | — | Most values were below the reporting limit |
| B. IL-17 detectability and availability by group | IL-17 | RA | 0.00 [0.00–0.00] | 6/89 (6.7%) | — | — | Low detectability |
|  | IL-17 | CT-RA | NA | Not available | — | — | Not comparable with controls |
|  | IL-17 | SLE | 0.00 [0.00–0.00] | 6/75 (8.0%) | — | — | Low detectability |
|  | IL-17 | CT-SLE | NA | Not available | — | — | Not comparable with controls |
|  | IL-17 | SpA | 0.00 [0.00–0.00] | 1/57 (1.8%) | — | — | Low detectability |
|  | IL-17 | CT-SpA | NA | Not available | — | — | Not comparable with controls |
| C. IL-10 case-control comparisons | IL-10 | RA vs CT-RA | 0.00 [0.00–0.00] vs 0.00 [0.00–0.00] | 10/89 (11.2%) vs 18/89 (20.2%) | 0.061 | 0.149 | No statistically significant difference detected |
|  | IL-10 | SLE vs CT-SLE | 0.00 [0.00–0.00] vs 0.00 [0.00–0.00] | 3/75 (4.0%) vs 1/76 (1.3%) | 0.300 | 0.367 | No statistically significant difference detected |
|  | IL-10 | SpA vs CT-SpA | 0.00 [0.00–0.00] vs 0.00 [0.00–0.00] | 3/57 (5.3%) vs 10/56 (17.9%) | 0.040 | 0.043 | Nominally lower IL-10 in SpA; interpret cautiously due to low detectability |
| D. IL-17 case-control comparability | IL-17 | RA vs CT-RA | 0.00 [0.00–0.00] vs NA | 6/89 (6.7%) vs Not available | — | — | Not formally compared because control data were unavailable or insufficient |
|  | IL-17 | SLE vs CT-SLE | 0.00 [0.00–0.00] vs NA | 6/75 (8.0%) vs Not available | — | — | Not formally compared because control data were unavailable or insufficient |
|  | IL-17 | SpA vs CT-SpA | 0.00 [0.00–0.00] vs NA | 1/57 (1.8%) vs Not available | — | — | Not formally compared because control data were unavailable or insufficient |

*Notes: This online resource summarizes serum IL-10 and IL-17 detectability and case-control comparisons. Values below the reporting limit were recorded as 0. Detectability was defined as values greater than 0. Continuous cytokine values were compared using Mann–Whitney U tests, and detectability proportions were compared using Fisher’s exact tests. IL-10 was detectable in a small proportion of samples in all groups, and most values were below the reporting limit. IL-17 results are reported descriptively only for patient groups because comparable control data were unavailable; therefore, IL-17 was not formally compared between cases and controls. Cytokine results should be interpreted as exploratory and descriptive rather than as robust mechanistic evidence.*
